# Diverse antimicrobial interactions of halophilic archaea and bacteria extend over geographical distances and cross the domain barrier

**DOI:** 10.1002/mbo3.115

**Published:** 2013-08-08

**Authors:** Nina S Atanasova, Maija K Pietilä, Hanna M Oksanen

**Affiliations:** Institute of Biotechnology and Department of Biosciences, University of HelsinkiViikinkaari 5, PO Box 56, 00014, Helsinki, Finland

**Keywords:** Antimicrobial substances, halocins, halophilic archaea, halophilic bacteria, hypersaline.

## Abstract

The significance of antimicrobial substances, halocins, produced by halophilic archaea and bacteria thriving in hypersaline environments is relatively unknown. It is suggested that their production might increase species diversity and give transient competitive advances to the producer strain. Halocin production is considered to be common among halophilic archaea, but there is a lack of information about halocins produced by bacteria in highly saline environments. We studied the antimicrobial activity of 68 halophilic archaea and 22 bacteria isolated from numerous geographically distant hypersaline environments. Altogether 144 antimicrobial interactions were found between the strains and aside haloarchaea, halophilic bacteria from various genera were identified as halocin producers. Close to 80% of the interactions were detected between microorganisms from different genera and in few cases, even across the domain boundary. Several of the strains produced halocins with a wide inhibitory spectrum as has been observed before. Most of the antimicrobial interactions were found between strains from distant sampling sites indicating that hypersaline environments around the world have similar microorganisms with the potential to produce wide activity range antimicrobials.

## Introduction

Extremely halophilic archaea from the family *Halobacteriaceae* are the dominating microorganisms in hypersaline environments, that is, solar salterns and natural salt lakes, at NaCl concentrations close to saturation (Oren 2002a; Sabet et al. 2009). Such environments are found on all continents and several studies illustrate that their microbiota is fairly similar across long spatial distances (Litchfield and Gillevet 2002; Oren 2002b; Atanasova et al. 2012). Aside haloarchaea, diverse bacterial communities including members of *Bacteroidetes* and gamma proteobacteria can be found at intermediate salinities (∼20% w/v NaCl) and *Salinibacter ruber* is abundant even at saturated levels of NaCl (∼35% w/v) (Antón et al. 2000; Oren 2002a,b; Ventosa 2006; Ghai et al. 2011). Some eukaryotes, such as the green algae *Dunaliella salina*, can also tolerate salt concentrations close to saturation, but generally eukaryotes thrive at lower salinities (Oren 2008). Bacterivory due to halophilic protozoa, ciliates, and flagellates is considered insignificant to the mortality of extremely halophilic microorganisms (Oren 2008). In the highest NaCl concentrations, microbial predators are nearly absent or represented by members of a single species (Hauer and Rogerson 2005). Viruses on the other hand are abundant in hypersaline environments and presumably have an effect on the ecology of their host cells as well as on the cycling of nutrients (Guixa-Boixareu et al. 1996; Rodríguez-Valera et al. 2009; Roine and Oksanen 2011; Atanasova et al. 2012).

When competing about life space and nutrients, organisms of all three domains of life are known to produce proteinaceous antagonists, which can inhibit the growth of closely related strains (Tagg et al. 1976; Riley and Wertz 2002). Several bacteriocins and eukaryocins have been characterized in detail (O'Connor and Shand 2002). Inhibitory substances produced by archaea, “archaeocins”, were first detected among extreme halophiles in 1982 and were given the name halocins (Rodríguez-Valera et al. 1982). Later, similar substances were identified among the crenarchaeal *Sulfolobales* cells (sulfolobicins) (Prangishvili et al. 2000). Here, the word halocin is used to describe the antimicrobial substances produced by both halophilic archaea and bacteria. To date, all the described halocins are produced by halophilic archaea from the family *Halobacteriaceae* as secreted compounds (Shand and Leyva 2008). Only a few halocins have been subjected to a more detailed study, but it seems that hundreds of different types exist (Shand and Leyva 2008). Halocins are divided into protein halocins and microhalocins based on their molecular mass. Protein halocins include H1 and H4, with a size range of approximately 30–40 kDa (Meseguer and Rodríguez-Valera 1985; Shand and Leyva 2007). Microhalocins, such as H6/H7, R1, C8, S8, and U1, are smaller than 10 kDa (Shand and Leyva 2007) except for Sech7a, which is 10.7 kDa (Pašić et al. 2008). The described microhalocins are characterized by the ability to withstand low salt concentrations, heating, and long-term storage while protein halocins are generally more sensitive to environmental stress (Meseguer and Rodríguez-Valera 1985; Shand and Leyva 2007; Pašić et al. 2008). Although several studies have illustrated that halocin production is widely spread among different halophilic archaea (O'Connor and Shand 2002; Shand and Leyva 2008), the well described halocins are limited to those produced by certain strains of *Haloferax* and *Halobacterium* or some uncharacterized members of *Halobacteriaceae* (Meseguer et al. 1986; Torreblanca et al. 1994).

Bacteriocins are known to have several different inhibitory mechanisms depending on the producer strain. Most of these functions target the cell membrane of sensitive strains (Nissen-Meyer and Nes 1997). Pore formation or depolarization of the cell membrane, nuclease or anion carrier activity, spore outgrowth inhibition, or enzyme inhibition have been described to result from bacteriocin activity (Riley 1998; Moll et al. 1999; Riley and Wertz 2002). Little is known about the inhibitory functions of halocins. However, halocin H6/H7 produced by *Haloferax gibbonsii* has been shown to inhibit the growth of sensitive strains by targeting the Na^+^/H^+^ antiporter causing cell lysis (Meseguer et al. 1995). The mechanism of immunity to halocins was studied with halocin C8 from a *Halobacterium* strain, revealing that the halocin as well as its immunity protein are encoded by the same gene (Sun et al. 2005). For the known bacteriocins, these two functions are encoded by different genes that are usually cotranscribed (Fimland et al. 2002). The known halocins are encoded by genes in megaplasmids and in most cases the expression reaches its peak at the transition from exponential to stationary growth phase (Shand and Leyva 2007).

The inhibitory spectrum of halocins is often wide, extending to euryarchaea of different genera and in some cases, crenarchaea as well (O'Connor and Shand 2002). The role of halocin production in interspecies competition is yet uncertain but it has been suggested that this type of antimicrobials might play a role in maintaining species diversity in extremely halophilic environments (Torreblanca et al. 1994; Shand and Leyva 2007).

In our previous study, numerous virus–host interactions were found between geographically distant hypersaline environments highlighting the uniformity of this type of environments (Atanasova et al. 2012). Here, we extend our analysis to antagonistic interactions of the halophilic archaea and bacteria. Our results support the universal characteristics of halocin production and emphasize that halocins can function across species and domain boundaries illustrating the diverse interactions among microorganisms in hypersaline environments.

## Materials and Methods

### Archaeal and bacterial strains and growth media

Halophilic archaeal and bacterial strains used in this study are listed in Table 1. Halophilic strains were grown aerobically using modified growth medium (MGM) (http://www.haloarchaea.com/resources/halohandbook/) (Nuttall and Dyall-Smith 1993; Atanasova et al. 2012) at 37°C.

**Table 1:** Prokaryotic strains used in this study

Number	Strain	Domain^1^	Sample/Origin^2^	GenBank acc no. of partial 16S rRNA gene sequence	Reference
1	*Salicola* sp. PV3	B	Tra (saltern)	FJ042665	Kukkaro and Bamford (2009)
2	*Salicola* sp. PV4	B	Tra (saltern)	FJ042666	Kukkaro and Bamford (2009)
3	*Salicola* sp. s3-1	B	MdS (saltern)	JN196461	Atanasova et al. (2012)
4	*Salicola* sp. s3-2	B	MdS (saltern)	JN196462	Atanasova et al. (2012)
5	*Salicola* sp. E200-5	B	Eil (saltern)	JN196498	Atanasova et al. (2012)
6	*Halogranum* sp. PV5	A	Tra (saltern)	JN196457	Atanasova et al. (2012)
7	*Pontibacillus* sp. PV1	B	Tra (saltern)	JN196455	Atanasova et al. (2012)
8	*Pontibacillus* sp. SP9-4	B	SP (experimental Dead Sea-Red Sea saltwater ponds)	JN196491	Atanasova et al. (2012)
9	*Pontibacillus* sp. SL-1	B	SL (saltern)	JN196514	Atanasova et al. (2012)
10	*Halomonas* sp. PV2	B	Tra (saltern)	JN196456	Atanasova et al. (2012)
11	*Halomonas* sp. s1e-1	B	MdS (saltern)	JN196460	Atanasova et al. (2012)
12	*Haloferax* sp. s5a-1	A	MdS (saltern)	JN196464	Atanasova et al. (2012)
13	*Halomonas* sp. E200-1	B	Eil (saltern)	JN196494	Atanasova et al. (2012)
14	*Salinivibrio* sp. E200-2	B	Eil (saltern)	JN196495	Atanasova et al. (2012)
15	*Halorubrum* sp. s1-1	A	MdS (saltern)	FJ042667	Kukkaro and Bamford (2009)
16	Halorubrum sp. SS5-4	A	SSB (saltern)	JN196482	Atanasova et al. (2012)
17	*Haloferax* sp. SP10-1	A	SP (experimental Dead Sea-Red Sea saltwater ponds)	JN196492	Atanasova et al. (2012)
18	*Chromohalobacter* sp. DS75-1	B	DS (lake)	JN196508	Atanasova et al. (2012)
19	*Chromohalobacter* sp. DS75-2	B	DS (lake)	JN196509	Atanasova et al. (2012)
20	*Chromohalobacter* sp. DS75-3	B	DS (lake)	JN196510	Atanasova et al. (2012)
21	*Chromohalobacter* sp. DS75-4	B	DS (lake)	JN196511	Atanasova et al. (2012)
22	*Chromohalobacter* sp. DS75-5	B	DS (lake)	JN196512	Atanasova et al. (2012)
23	*Haloarcula hispanica* ATCC 33960	A	Spain, Alicante (saltern)	U68541	Juez et al. (1986)
24	*Halomonas* sp. SS2-3	B	SSB (saltern)	JN196472	Atanasova et al. (2012)
25	*Halorubrum* sp. s1-2	A	MdS (saltern)	JN196459	Atanasova et al. (2012)
26	*Halorubrum* sp. s5a-3	A	MdS (saltern)	JN196466	Atanasova et al. (2012)
27	*Haloarcula marismortui* ATCC 43049	A	DS (lake)	X61688	Oren et al. (1990), Mylvaganam and Dennis (1992)
28	*Haloarcula quadrata* ATCC 700850	A	Egypt, Sinai (sabkha)	AB010964	Oren et al. (1999)
29	*“*Haloarcula sinaiiensis*”* ATCC 33800	A	Egypt, Sinai (sabkha)	D14129	Javor et al. (1982)
30	*Haloarcula vallismortis* ATCC 29715	A	USA, California, Death Valley (salt pool/lake)	AB355982	Gonzalez et al. (1978), Torreblanca et al. (1986)
31	*Halorubrum sp*. PV6	A	Tra (saltern)	FJ685652	Pietilä et al. (2009)
32	*Haloarcula* sp. PV7	A	Tra (saltern)	JN196458	Atanasova et al. (2012)
33	*Halorubrum* sp. s5a-2	A	MdS (saltern)	JN196465	Atanasova et al. (2012)
34	*Halorubrum* sp. B2-2	A	SSA (saltern)	JN196469	Atanasova et al. (2012)
35	*Halosarcina* sp. SS2-4	A	SSB (saltern)	JN196473	Atanasova et al. (2012)
36	*Halogeometricum* sp. SS4-3	A	SSB (saltern)	JN196477	Atanasova et al. (2012)
37	*Halogranum* sp. SS5-1	A	SSB (saltern)	JN196479	Atanasova et al. (2012)
38	*Halorubrum* sp. SL-5	A	SL (saltern)	JN196518	Atanasova et al. (2012)
39	*Halogeometricum* sp. CG-6	A	CG (saltern)	JN196533	Atanasova et al. (2012)
40	*Halogeometricum* sp. CG-12	A	CG (saltern)	JN196535	Atanasova et al. (2012)
41	*Halorubrum* sp. SS1-3	A	SSB (saltern)	JN196470	Atanasova et al. (2012)
42	*Salarchaeum* sp. SL-3	A	SL (saltern)	JN196516	Atanasova et al. (2012)
43	*Halosarcina* sp. GV-8	A	GV (saltern)	JN196527	Atanasova et al. (2012)
44	*Halorubrum sodomense* DSM 33755	A	DS (lake)	D13379	Oren (1983)
45	“Haloarcula californiae” ATCC 33799	A	Mexico, Baja California (brine pool)	AB477984	Javor et al. (1982)
46	*Haloarcula japonica* TR1 ATCC 49778	A	Japan, Noto Peninsula (brine pool)	NR_028234	Takashina et al. (1990)
47	*Halorubrum* sp. s3-3	A	MdS (saltern)	JN196463	Atanasova et al. (2012)
48	*Halorubrum* sp. s5a-4	A	MdS (saltern)	JN196467	Atanasova et al. (2012)
49	*Halorubrum* sp. s5a-6	A	MdS (saltern)	JN196468	Atanasova et al. (2012)
50	*Halorubrum* sp. SS2-6	A	SSB (saltern)	JN196474	Atanasova et al. (2012)
51	*Halogeometricum* sp. SS4-4	A	SSB (saltern)	JN196478	Atanasova et al. (2012)
52	*Haloarcula* sp. SS5-2	A	SSB (saltern)	JN196480	Atanasova et al. (2012)
53	*Halosarcina* sp. SS5-3	A	SSB (saltern)	JN196481	Atanasova et al. (2012)
54	*Halosarcina* sp. SS5-5	A	SSB (saltern)	JN196483	Atanasova et al. (2012)
55	*Halorubrum* sp. SS5-7	A	SSB (saltern)	JN196484	Atanasova et al. (2012)
56	*Halorubrum* sp. SS5-8	A	SSB (saltern)	JN196485	Atanasova et al. (2012)
57	*Halorubrum* sp. E200-3	A	Eil (saltern)	JN196496	Atanasova et al. (2012)
58	*Halorubrum* sp. E200-4	A	Eil (saltern)	JN196497	Atanasova et al. (2012)
59	*Haloarcula* sp. E200-6	A	SP (experimental Dead Sea-Red Sea saltwater ponds)	JN196488	Atanasova et al. (2012)
60	*Halorubrum* sp. E301-2	A	Eil (saltern)	JN196500	Atanasova et al. (2012)
61	*Haloarcula* sp. E301-5	A	Eil (saltern)	JN196503	Atanasova et al. (2012)
62	*Halorubrum* sp. E302-1	A	Eil (saltern)	JN196504	Atanasova et al. (2012)
63	*Haloarcula* sp. E303-4	A	Eil (saltern)	JN196507	Atanasova et al. (2012)
64	*Halobacteriaceae* sp. SL-2	A	SL (saltern)	JN196515	Atanasova et al. (2012)
65	*Halorubrum* sp. GV-4	A	GV (saltern)	JN196523	Atanasova et al. (2012)
66	*Halogranum* sp. CG-2	A	CG (saltern)	JN196530	Atanasova et al. (2012)
67	*Halorubrum* sp. CG-4	A	CG (saltern)	JN196532	Atanasova et al. (2012)
68	*Halogeometricum* sp. CG-9	A	CG (saltern)	JN196534	Atanasova et al. (2012)
69	*Halorubrum* sp. E301-4	A	Eil (saltern)	JN196502	Atanasova et al. (2012)
70	*Halobacterium* sp. SL-4	A	Eil (saltern)	JN196499	Atanasova et al. (2012)
71	*Halobacterium* sp. SL-6	A	SL (saltern)	JN196519	Atanasova et al. (2012)
72	*Halogeometricum* sp. CG-3	A	CG (saltern)	JN196531	Atanasova et al. (2012)
73	*Salisaeta* sp. SP10-4	B	SP (experimental Dead Sea-Red Sea saltwater ponds)	JN196493	Atanasova et al. (2012)
74	*Halogeometricum* sp. GV-7	A	GV (saltern)	JN196526	Atanasova et al. (2012)
75	*Halorubrum* sp. E301-3	A	Eil (saltern)	JN196501	Atanasova et al. (2012)
76	*Halorubrum* sp. E303-2	A	Eil (saltern)	JN196506	Atanasova et al. (2012)
77	*Halosarcina* sp. CG-1	A	CG (saltern)	JN196529	Atanasova et al. (2012)
78	*Halorubrum* sp. SS3-5	A	SSB (saltern)	JN196476	Atanasova et al. (2012)
79	*Halorubrum* sp. SP3-3	A	SP (experimental Dead Sea-Red Sea saltwater ponds)	JN196487	Atanasova et al. (2012)
80	*Haloarcula* sp. E303-1	A	Eil (saltern)	JN196505	Atanasova et al. (2012)
81	*Halorubrum* sp. GV-9	A	GV (saltern)	JN196528	Atanasova et al. (2012)
82	*Halobacterium* sp. SL-7	A	SL (saltern)	JN196520	Atanasova et al. (2012)
83	*Halorubrum* sp. GV-6	A	GV (saltern)	JN196525	Atanasova et al. (2012)
84	*Halobacteriaceae* sp. SP3-2	A	SP (experimental Dead Sea-Red Sea saltwater ponds)	JN196486	Atanasova et al. (2012)
85	*Rhodovibrio* sp. GV-2	B	GV (saltern)	JN196521	Atanasova et al. (2012)
86	*Rhodovibrio* sp. GV-3	B	GV (saltern)	JN196522	Atanasova et al. (2012)
87	*Haloplanus* sp. SP5-1	A	SL (saltern)	JN196517	Atanasova et al. (2012)
88	*Salisaeta* sp. SP9-1	B	SP (experimental Dead Sea-Red Sea saltwater ponds)	JN196489	Atanasova et al. (2012)
89	*Halorubrum* sp. SP9-2	A	SP (experimental Dead Sea-Red Sea saltwater ponds)	JN196490	Atanasova et al. (2012)
90	*Natronomonas* sp. GV-5	A	GV (saltern)	JN196524	Atanasova et al. (2012)

1 A, Archaea; B, Bacteria

2 Tra, Trapani, Sicily, Italy; MdS, Margherita di Savoia, Italy; SSA, Samut Sakhon, Thailand 2007; SSB, Samut Sakhon, Thailand 2008; SP, Sedom Ponds Israel; Eil, Eilat Israel; DS, The Dead Sea, Israel; SL, Sečovlje, Slovenia; GV, Guardias Viejas, Spain; CG Cabo de Gata, Spain.

### Production of culture supernatants and antimicrobial activity assay

Culture supernatants of the halophilic strains (Table 1) were prepared for antimicrobial activity tests by removing cells from early stationary phase cultures (OD_550_ = 0.3–1.3) by centrifugation (Heraeus Biofuge, 15 700 *g*, 5 min, 22°C). All preparations used for antimicrobial screening were stored at 4°C for no longer than 4 months.

For antimicrobial activity tests, 500 μL of indicator strain (Table 1) in early exponential phase (OD_550_ = 0.3-0.8) was mixed with 3 mL of MGM soft agar (55°C), spread on an MGM plate, and incubated at 22°C for 1 h. The slowest growing strains, *Rhodovibrio* sp. GV-2, *Rhodovibrio* sp. GV-3, *Natronomonas* sp. GV-5, *Halorubrum* sp. GV-6, and *Haloplanus* sp. SP5-1 (strains 85, 86, 90, 83, and 87), were plated when OD_550_ was 0.5, 0.5, 0.4, 0.4, and 0.3, respectively. After the 1-h incubation, 10 μL of culture supernatants were spotted on the plates containing the indicator strains. MGM broth was used as a control. The plates were incubated at 22°C for 1 h followed by incubation at 37°C until the indicator strain was well grown as a lawn or when the inhibitory zones appeared (incubation times were from 1 day to 2 weeks depending on the growth rate of the indicator strains). The diameters of the inhibition zones were measured when the zones reached the maximal size.

### Sensitivity of the produced halocins to proteases

Culture supernatants of halocin producers were tested for protease sensitivity using trypsin (Gibco) and proteinase K (Roche) (2 mg/mL final concentration; the stock solutions being 20 mg/mL in 23% salt water buffer (http://www.haloarchaea.com/resources/halohandbook/)). The culture supernatants were treated with the protease for 1 h at 37°C after which protease activity was blocked by addition of protease inhibitor (Complete Mini EDTA-free Protease Inhibitor Cocktail Tablets, Roche). After incubation (1 h at 22°C), the halocin activity of the culture supernatants was determined by antimicrobial activity assay.

### Virus isolation and analysis

Dilution series (10^0,^ 10^−2^, 10^−4^, 10^−6^) of culture supernatants obtained from early stationary growth phase were spotted on indicator strain lawns. The plates were incubated as mentioned above. Single plaques were picked from spots containing diluted culture supernatant, and plaque-purified three consecutive times. Virus stocks were prepared, viruses purified, and negative staining electron microscopy was performed as described in (Atanasova et al. 2012).

### Phylogenetic analysis

The phylogenetic analysis of the strains was performed by the maximum likelihood method with the Tamura and Nei substitution model for nucleotide sequences using the Molecular Evolutionary Genetics Analysis (MEGA) software version 5 (Tamura et al. 2011). The analysis was evaluated by 1000 bootstrap samplings.

## Results

### Halocin production was a common phenomenon for halophilic archaea and bacteria

We studied the antimicrobial production of previously isolated halophilic strains including 60 archaea and 22 bacteria, (Atanasova et al. 2012) and eight archaeal culture collection strains numbered from one to 90 in this investigation (Table 1). In the text, the strain numbers are also indicated after the names. The archaeal and bacterial strains have been previously isolated from nine spatially distant hypersaline environments including different solar salterns, experimental ponds at Sedom, Israel, and the Dead Sea (Atanasova et al. 2012; Table 1). Antimicrobial activity was assayed by using culture supernatants of the producer strains in early stationary growth phase. The supernatants were tested with exponentially growing indicator organisms, all strains against all (Table 2).

**Table 2:** Halocin production and sensitivity of the halophilic archaeal and bacterial strains

Producers^1^	1	2	3	4	5	6	8	9	12	14	17	24	34	36	38	40	41	44	47	48	50	52	54	55	57	58	66	67	68	69	70	75	76	78	87	89
Indicators																																				
6									8																											
8	13^2^	15	14		13				19				11				14				12			9			15									
9	12	11	11	13	8				16				10				11										13									
15									7																					9						
16									9																											
19		7																																		
22												5																								
24																				7																
25			15						10																											
26									6																											
27									12																											
28									21																											
29									8																											
30									7													10														
31									7																											
34									23																											
36									12																											
37									7																											
39									12																											
40									5										10														11			10
41									7				15		12			12																		
42									8																		7									
43									7																											
44									5																	12										
45									14													13														
46									13																											
47									7																											
48									13																											
49									16																											
50									9																	12										
51									14																								11	5		
53									20					10	12																					
54									19																											
55									6						12	11																				
56									5																											
57									6																											
58									4																											
59									12																											
60									7																											
61									6																											
62									9																	13										
63									12																											
64																									14	12										
65									9																16	20				13						
66						12			20																	9										
67									8																											
69									9																											
70									8																					8						
71									12																											
72									8																											
74									10								6																			
75									13																											
76									10							7									6	7				9						
77									15																											
78									16																12	13				9						
79									8																13	13		11		7		7				
80									21																				10	9			13		8	14
81									30		11											10	15		10	12				9						
82									13																						13					
84									25																				20							
85											12																									
86											12																									
88							12	13		11							9		9							11										
89									20																25	29				23						
90									20		14																									

1 Strains (See Table 1).

2 Diameter of the inhibitory zone (mm).

**Table 3:** Reference strain accession numbers used in the 16S rRNA gene comparison

Strain	GenBank Accession Number
*Halorubrum aidingense*	DQ355813
*Halorubrum lipolyticum*	DQ355814
*Halorubrum saccharovorum*	X82167
*Halorubrum lacusprofundi*	X82170
*Halorubrum trapanicum*	X82168
*Haloplanus natans*	AB477975
*Haloplanus vescus* RO5-8T	EU931578
*Haloplanus aerogenes*	GQ282625
*Halogranum gelatinilyticum*	GQ282624
*Halogranum rubrum*	EU887283
*Halogranum amylolyticum*	GQ282623
*Haloferax volcanii*	K00421
*Halosarcina pallida*	AB477980
*Halogeometricum borinquense*	AF002984
*Natronomonas pharaonis*	D87971
*Salarcheum japonicum*	AB454051
*Halobacterium jilantaiense*	AB477970
*Halobacterium noricense*	NR_028187
*Pontibacillus chungwhensis*	AY553296
*Salisaeta longa*	EU426570
*Rhodovibrio salinarum*	M59069
*Salinivibrio costicola*	NR_027590
*Salicola salis*	DQ129689
*Salicola marasensis*	DQ019934
*Halomonas meridiana*	AJ306891
*Halomonas halmophila*	M59153
*Halomonas elongata*	X67023
*Halomonas shengliensis*	EF121853
*Chromohalobacter marismortui*	X87219
*Chromohalobacter canadensis*	AJ295143
*Chromohalobacter salexigens*	AJ295146

**Figure 1:**
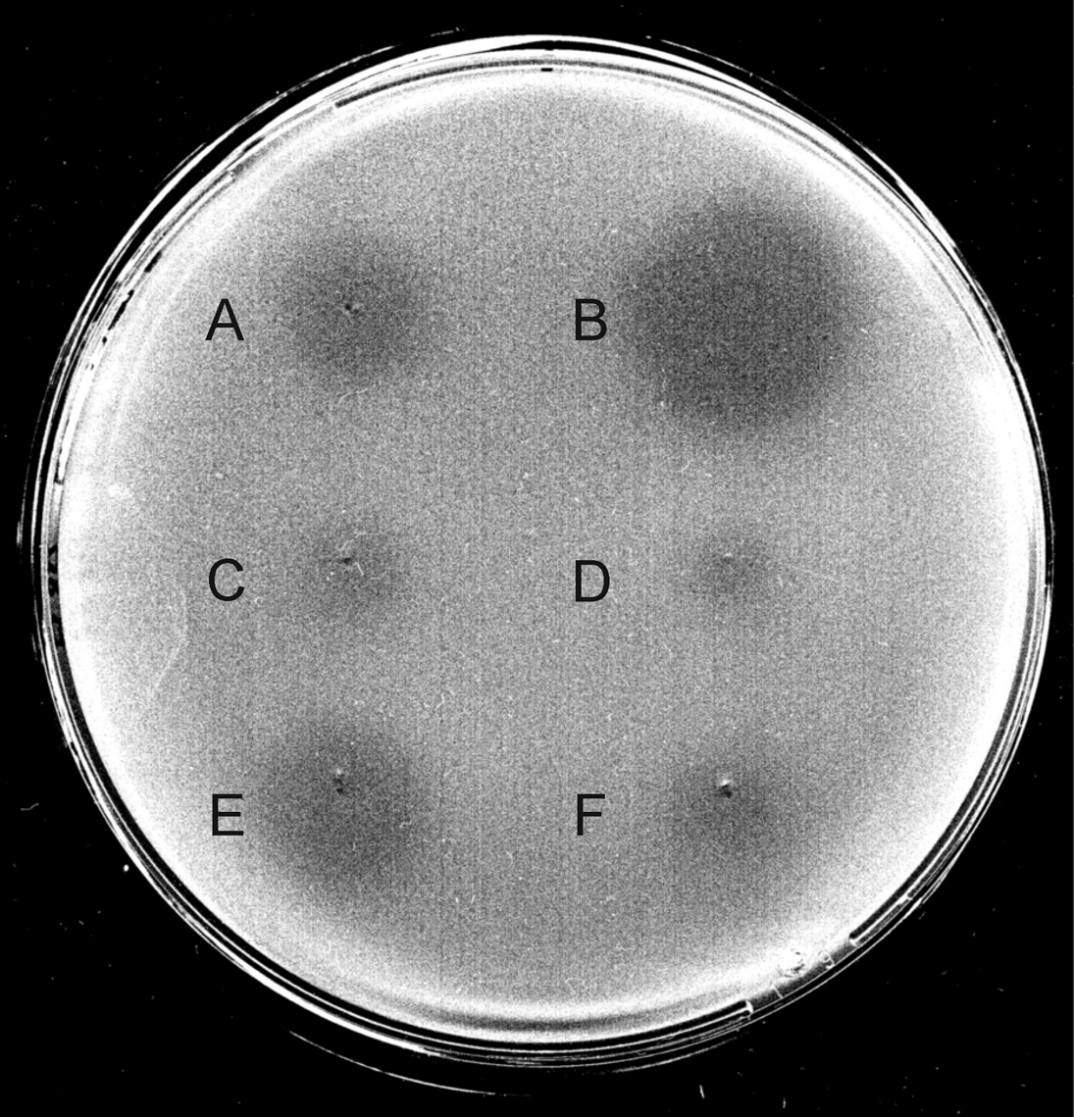
Detection of halocins. The activity of halocins produced by (A) *Halogeometricum* sp. CG-9, (B) *Haloferax* sp. s5a-1, (C) *Halorubrum* sp. E303-2, (D) *Halorubrum* sp. E301-4, (E) *Halorubrum* sp. SP9-2, and (F) *Haloplanus* sp. SP5-1, on the lawn of the indicator strain *Haloarcula* sp. E303-1. For visualization, the culture supernatants were filtrated (Sartorius Stedim Minisart High-Flow, pore size 0.20 μm).

The production of halocins was observed as growth inhibitory zones (Fig. 1), which could be a result of either lysis of the cells (cytocidal effect) or cell growth inhibition (cytostatic effect). For most halocins, inhibition was only detectable in the undiluted sample. The diameters of the inhibitory zones varied from four to 30 mm (Table 2; Fig. 1), which suggests that the produced halocins might be different molecules. In several cases, a group of strains was inhibiting the growth of a single sensitive strain. To confirm that inhibition was not a result of virus infection, multiple dilutions of producer strain culture supernatants were applied on indicator lawns. Plaques were found when the 10^−2^ and 10^−4^ diluted supernatants of *Halorubrum* sp. SS5-4 (strain 16) and *Halorubrum* sp. B2-2 (strain 34) were tested with *Halorubrum* sp. SS5-4 (strain 16). Those were confirmed to be viruses by plaque assay and negative stain electron microscopy of purified virions (data not shown).

The cross-testing of all the 90 strains revealed 144 halocin production-sensitivity interactions (Table 2). Altogether 36 strains (27 archaea and nine bacteria) were producing halocins against 65 sensitive strains (57 archaea and eight bacteria). No strain was shown to be sensitive to its own halocin(s). Only 13 strains (six archaeal and seven bacterial) did not participate in the halocin production-sensitivity network. These strains were resistant to all the halocins and did not produce inhibitory substances against any indicator organism.

A broad inhibition range was characteristic to many halocin-producing strains (Table 2). Culture supernatants of six producers inhibited the growth of four or more indicators. *Haloferax* sp. s5a-1 (strain 12) halocin(s) had the widest activity spectrum inhibiting 58 different strains. The other halocins with a broad inhibitory effect were from *Halorubrum* sp. E200-4 (strain 58) (12 sensitive strains), *Halorubrum* sp. E301-4 (strain 69) (nine sensitive strains), *Halorubrum* sp. E200-3 (strain 57) (seven sensitive strains), *Haloferax* sp. SP10-1 (strain 17) (four sensitive strains), and *Halorubrum* sp. SS1-3 (strain 41) (four sensitive strains). Halocins of 13 producers inhibited the growth of two or three sensitive strains. The rest of the producers (17 in total) inhibited only a single indicator. Broad inhibition was characteristic only to the archaeal halocin producers as bacterial halocins inhibited the growth of only one or two strains.

In many cases, several producer strains inhibited the growth of a single indicator. As an example, halocins produced by a set of nine strains (Table 2) were active against *Pontibacillus* sp. SP9-4 (strain 8). Eight and seven strains, respectively, inhibited the growth of *Pontibacillus* sp. SL-1 (strain 9) and *Halorubrum* sp. GV-9 (strain 81) (Table 2). The other sensitive strains were targeted by one to six different producers.

### Halocin production and sensitivity were distributed widely among archaeal and bacterial taxa

**Figure 2:**
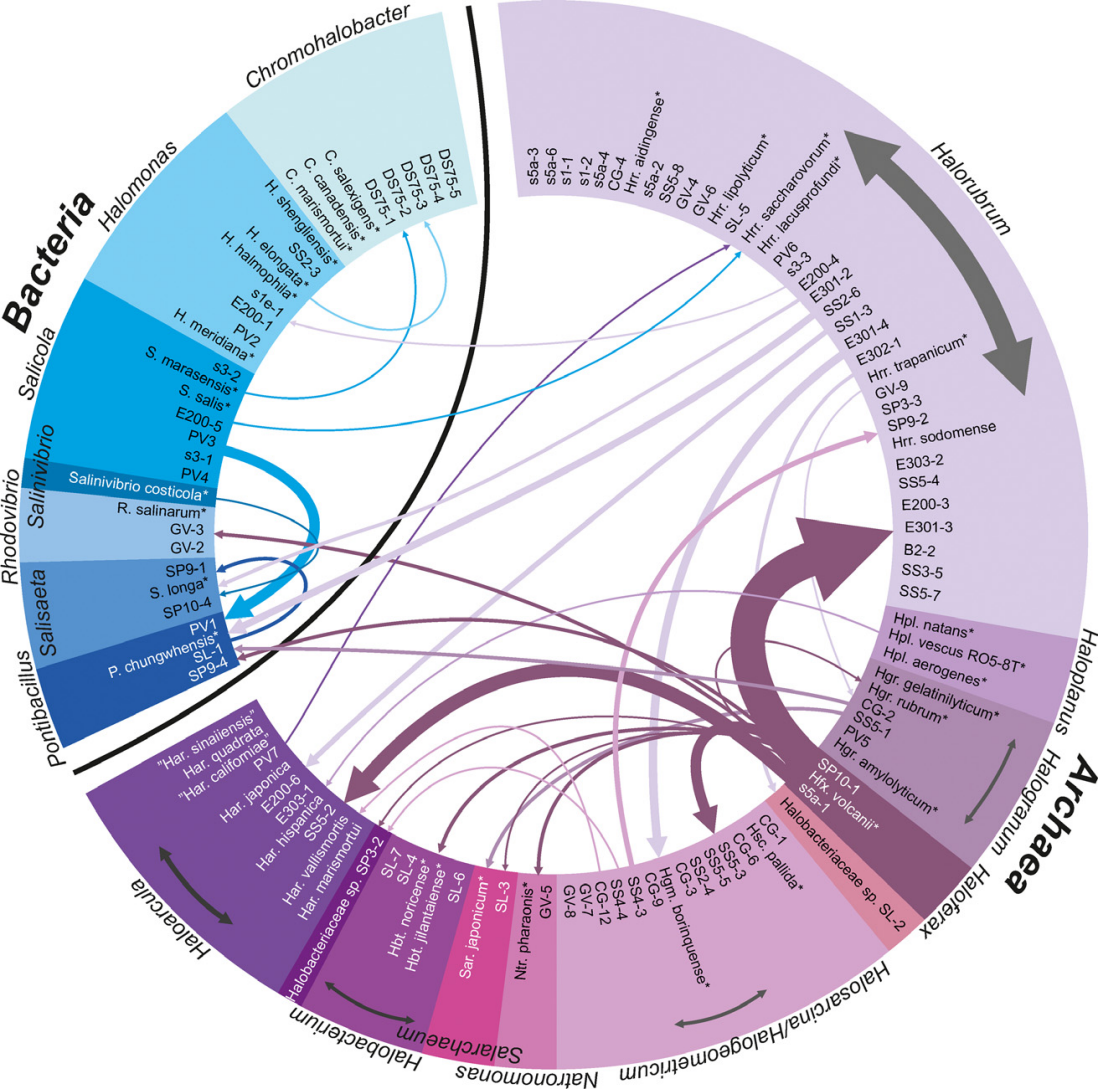
Halocin activity across and within genera. Phylogenetic analysis of 16S rRNA gene sequences of the 90 strains used in the study is based on maximum likelihood. Reference strains (marked with asterisks) are included in the phylogenetic analysis and their GenBank accession numbers are listed in Table 3. The maximum likelihood tree is removed from the picture for clarity, but the grouping of the strains is the same as shown in Atanasova et al. 2012. Archaeal and bacterial domains are separated by a space and a black line and the strains are grouped into colored sectors according to their genus. The two strains of *Halobacteriaceae* sp., SL-2 and SP3-2, with no determined genus are grouped in separate sectors. Strains belonging to the two genera, *Halosarcina* and *Halogeometricum*, are combined in the same group due to their close relatedness. The arrows in the sectors describe the halocin production-sensitivity interactions within one genus. The arrows in the central area describe the activity of halocin producers from one genus against strains in another genus. The thickness of the arrow relates to the amount of halocin production-sensitivity interactions between two genera or within one genus (see Table 4).

Based on the 16S rRNA gene sequences (Atanasova et al. 2012; Table 1), the studied 68 archaea and 22 bacteria belong to 17 genera. Halocin producers were identified in 12 different genera, eight archaeal and four bacterial (Fig. 2; Table 4). The sensitive strains were distributed into nine archaeal and five bacterial genera. Halocin sensitivity was more frequently observed between two strains belonging to different genera than within one genus (Fig. 2; Table 4). One hundred and eleven (77%) out of the 144 production-sensitivity interactions were detected between halocin producers and sensitive strains from different genera, and only 33 halocin interactions occurred within one genus. The majority (28 out of 33) of such interactions were observed among *Halorubrum* isolates. Sixteen out of the 30 *Halorubrum* strains were confirmed to produce halocins and 27 were halocin sensitive. Except for the strain *Halorubrum* sp. SL-5 (strain 38), all halocin-producing *Halorubrum* strains were also sensitive to these substances. Altogether, different *Halorubrum* strains inhibited the growth of microorganisms belonging to nine groups, including three bacterial genera.

The genus *Haloferax* was represented only by two isolates, *Haloferax* sp. s5a-1 and *Haloferax* sp. SP10-1 (strains 12 and 17), with exceptionally broad inhibitory spectra (Fig. 2; Table 4). Notably, neither one of the *Haloferax* isolates was inhibited by any of the halocins. The two strains belonging to *Natronomonas* and *Salarchaeum* (strains 90 and 42) and the two *Halobacteriaceae* isolates (strains 64 and 84) did not produce halocins, but were sensitive to halocins produced by other strains. In addition to the two *Haloferax* strains (strains 12 and 17), the only *Haloplanus* strain SP5-1 (strain 87) was not sensitive to any halocins.

Both gram-negative and gram-positive bacteria were identified as producers and sensitive strains (Fig. 2; Table 4). Most of the bacterial strains involved in halocin interactions were gamma proteobacteria. Others belonged to the phyla *Bacteroidetes* and *Firmicutes*. The nine bacterial halocin producers represented four genera (*Halomonas*, *Salicola*, *Pontibacillus*, and *Salinivibrio*). Six strains from five genera (*Chromohalobacter*, *Halomonas*, *Rhodovibrio*, *Salisaeta*, and *Pontibacillus*) were found to be sensitive to halocins. Three of the sensitive strains were also producers. The *Pontibacillus* strains (strains 8 and 9) represented the only gram-positive isolates participating in the halocin interactions.

**Table 4:** The number of halocin producer-sensitivity interactions between strains in different genera^1^

	Genus of the producers										
	
Genus of the sensitive indicator strains	*Halorubrum* (16)	*Haloarcula* (1)	*Halosarcina/Halogeometricum* (4)	*Halobacterium* (1)	*Halogranum* (2)	*Haloferax* (2)	*Haloplanus* (1)	*Halomonas* (1)	*Salicola* (5)	*Pontibacillus* (2)	*Salinivibrio* (1)
*Halorubrum* (27)^2^	**28**^3^	1	3			28			1		
*Haloarcula* (10)	3	**2**	1			10	1				
*Halosarcina/Halogeometricum* (10)	7		**1**			10					
*Halobacterium* (3)	1			**1**		3					
*Halogranum* (3)	1				**1**	3					
*Salarcheum* (1)					1	1					
*Halobacteriaceae* sp. SL-2 (1)	2										
*Halobacteriaceae* sp. SP3-2 (1)			1			1					
*Natronomonas* (1)						2					
*Chromohalobacter* (2)								1	1		
*Halomonas* (1)	1^4^										
*Rhodovibrio* (2)						2					
*Salisaeta* (1)	3									2	1
*Pontibacillus* (2)	6				2	2			9		
Total amount of interactions	52	3	6	1	4	62	1	1	11	2	1

1 Bacterial genera in gray.

2 The number of interacting strains in the genus.

3 Interactions within the same genus are in bold.

4 Cross domain interactions are underlined.

Halocin production and sensitivity across the domain border of *Archaea* and *Bacteria* were commonly observed (Fig. 2; Table 4). In 16 cases bioactive compounds of an archaeal strain were inhibiting the growth of a bacterial strain. Archaeal strains belonging to the genera *Halorubrum*, *Haloferax,* or *Halogranum* inhibited bacteria in the genera *Halomonas*, *Rhodovibrio*, *Salisaeta,* or *Pontibacillus*. Only one bacterial strain, *Salicola* sp. s3-1 (strain 3), produced a halocin(s) inhibiting the growth of an archaeal isolate, *Halorubrum* sp. s1-2 (strain 25) (Fig. 2; Table 4).

### The studied halocins were diverse in their sensitivity to proteases

In order to test the proteinaceous nature of halocins, a subset of eight halocin producers and 14 sensitive strains were chosen for the analysis (Table 5). The selected group included halocins that were active on archaeal and bacterial strains, including *Haloferax* sp. s5a-1 (strain 12) producing halocins with a broad inhibitory spectrum. Culture supernatants of the halocin producers were treated with proteinase K or trypsin followed by protease inhibitor treatment prior to the activity assay.

**Table 5:** Protease sensitivity of halocins

		Halocin sensitivity to	
		
Halocin-producing strain	Indicator strain	Proteinase K	Trypsin
*Salicola* sp. s3-1 (strain 3)	*Halorubrum* sp. s1-2 (strain 25)	+^1^	−2
*Haloferax* sp. s5a-1 (strain 12*)*	*Pontibacillus* sp. SP9-4 (strain 8)	−	−
*Haloferax* sp. s5a-1 (strain 12*)*	*Pontibacillus* sp. SL-1 (strain 9)	−	−
*Haloferax* sp. s5a-1 (strain 12*)*	*Halorubrum* sp. s1-1 (strain 15)	−	−
*Haloferax* sp. s5a-1 (strain 12*)*	*Halorubrum* sp. SS5-4 (strain 16)	−	−
*Haloferax* sp. s5a-1 (strain 12*)*	*Haloarcula quadrata* (strain 28)	+	−
*Haloferax* sp. s5a-1 (strain 12*)*	*Halorubrum* sp. B2-2 (strain 34)	+	−
*Haloferax* sp. s5a-1 (strain 12*)*	*Halorubrum* sp. s5a-4 (strain 48)	+	+
*Haloferax* sp. s5a-1 (strain 12*)*	*Halorubrum* sp. s5a-6 (strain 49)	+	+
*Haloferax* sp. s5a-1 (strain 12*)*	*Halorubrum* sp. SS3-5 (strain 78)	+	+
*Halorubrum sodomense* (strain 44)	*Halorubrum* sp. SS1-3 (strain 41)	+	−
*Haloarcula* sp. SS5-2 (strain 52)	*Haloarcula vallismortis* (strain 30)	+	−
*Haloarcula* sp. SS5-2 (strain 52)	*Haloarcula japonica* (strain 45)	+	−
*Halorubrum* sp. E200-3 strain 57)	*Halorubrum* sp. SS3-5 (strain 78)	+	−
*Halorubrum* sp. E200-4 (strain 58)	*Halorubrum* sp. SS3-5 (strain 78)	+	−
*Halorubrum* sp. E301-4 (strain 69)	*Halorubrum* sp. SS3-5 (strain 78)	+	+
*Halorubrum* sp. SS3-5 (strain 78)	*Halogeometricum* sp. SS4-4 (strain 51)	−	−

1 Sensitive to protease.

2 Resistant to protease.

The tested halocins could be divided into three groups based on their sensitivity to these proteases. Four halocins were sensitive to both proteases, five were resistant to both of them, and eight were sensitive to proteinase K but resistant to trypsin (Table 5). Halocins produced by *Haloferax* sp. s5a-1 (strain 12), active on nine sensitive strains, expressed properties of all three categories depending on the strain used in the assay. This indicates that the strain most probably produces several different halocins.

### Geographic distribution of halocin production-sensitivity interactions

**Figure 3:**
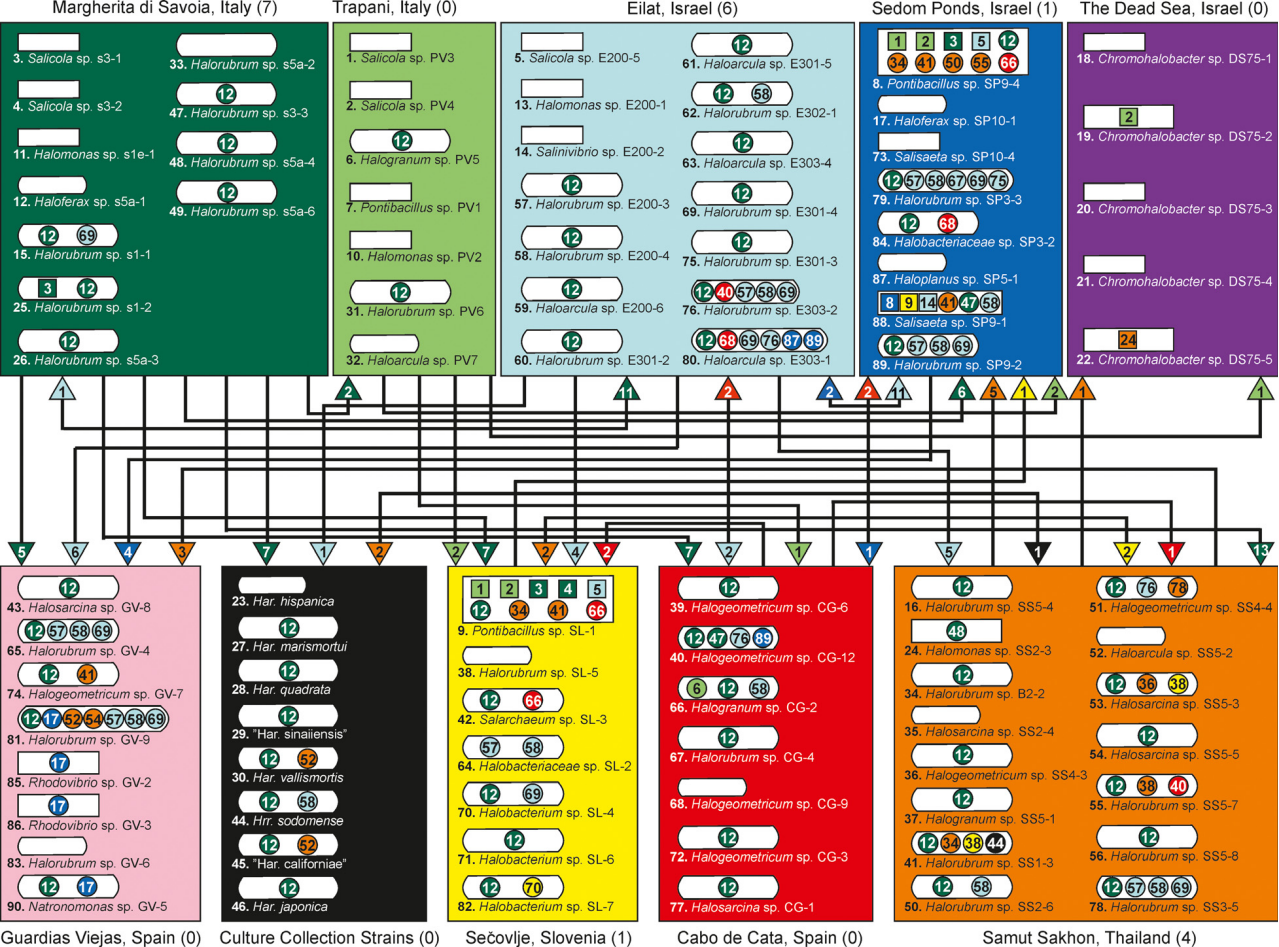
Halocin production and sensitivity of halophilic archaeal and bacterial strains according to the site of isolation. The large rectangles represent the isolation locations marked with specific colors (Atanasova et al. 2012; Table 1). The indicator organisms are shown by white rectangles with curved ends (archaea) or angular ends (bacteria). Active halocins produced by archaea and bacteria are indicated by circles and squares, respectively. The numerals and colors refer to the strain numbers and the origin of the producer, respectively. The arrows describe halocin production against strains isolated from different locations than the producer. The numbers inside the arrow heads refer to the number of sensitive strains in one location against the producers from another location. The numbers in parenthesis following location names indicate the number of sensitive strains against producers within one location.

Halocin production-sensitivity interactions were mapped in relation to the sampling site. Nineteen out of the 144 interactions were found between producers and sensitive strains isolated from the same sampling site (Fig. 3). Majority of the interactions (125 out of 144) were detected between producers and sensitive strains that were isolated from spatially distant locations (Fig. 3). This type of interactions was abundant in Sedom Ponds, Guardias Viejas, Sečovlje, and Samut Sakhon. In two locations, Margherita di Savoia and Trapani, most production-sensitivity interactions were due to the strain *Haloferax* sp. s5a-1 (strain 12). Although a relatively large number of strains originates from Margherita di Savoia, only one of the interactions was with a producer (*Halorubrum* sp. E301-4; strain 69) from a geographically distant environment.

The strains from the Dead Sea or Guardias Viejas were not found to produce halocins inhibiting the growth of the tested strains, although both locations contained halocin-sensitive strains (Fig. 3). In the culture collection strain group, only *Halorubrum sodomense* (strain 44) was identified as halocin producer. Bacterial halocins were active against strains from Margherita di Savoia, Sedom Ponds, the Dead Sea, Guardias Viejas, and Sečovlje. Archaeal halocins inhibited strains from all sites except for the bacterial strains from the Dead Sea.

## Discussion

Recent observations about the wide global interactions between extremely halophilic microorganisms and their viruses (Atanasova et al. 2012) let us to investigate the antagonistic interactions among these archaeal and bacterial strains (Table 1). Antimicrobial substances produced by halophilic archaea have been studied only for a few decades and the key element highlighted in research seems to be the universal characteristic of their production among different strains (Torreblanca et al. 1994).

In the past few studies, where a large set of haloarchaeal strains were tested against each other, halocin producers were found to be highly abundant and inhibiting strains belonging to different phylogenetic groups (Rodríguez-Valera et al. 1982; Meseguer et al. 1986; Torreblanca et al. 1994; Kis-Papo and Oren 2000). The number of sensitive strains was even higher and many were inhibited by several different halocins emphasizing that a broad inhibitory spectrum is characteristic to this type of antimicrobials. From our set of 90 halophilic archaea and bacteria, more than one third of the strains were identified as halocin producers and over two thirds were sensitive to these halocins. Often one strain was sensitive to several different halocins. Unlike in previous studies, none of our strains was inhibited by their own halocins. This phenomenon has been described before as a common feature for haloarchaea (Torreblanca et al. 1994).

Our test set differs from the previous studies by including strains belonging to halophilic bacterial genera. In several studied hypersaline environments halophilic bacteria have been documented to be abundant (Antón et al. 2000; Ghai et al. 2011; Atanasova et al. 2012) but hardly any information exists about their role as halocin producers. The results obtained here show that halophilic bacteria are producing diverse halocins that in some case can inhibit organisms from the archaeal domain as well (Table 2; Fig. 2). In fact, the most intriguing observation in this study was that halophilic archaea and bacteria are inhibiting each other across the domain barrier. Although most halocin studies have focused on the inhibitory interactions between different halophilic archaea, some evidence about cross-domain antimicrobial activity exist. It has been observed that halophilic archaea and bacteria can inhibit the growth of each other, and halophilic fungi can be antagonistic against both (Shand and Leyva 2007). In addition, some microhalocins have been shown to inhibit the growth of crenarchaeal *Sulfolobus* strains and some groups of pathogenic bacteria (O'Connor and Shand 2002; Shand and Leyva 2008; Kavitha et al. 2011).

Broad inhibitory spectra documented for several of the studied halocins distinguish them from bacteriocins, which are shown to inhibit only close relatives of the producing strain (O'Connor and Shand 2002; Riley and Wertz 2002). Bacteriocins of lactic acid bacteria, however, seem to represent another type of antimicrobials capable of inhibiting various types of microorganisms (De Vuyst and Leroy 2007). In our study, isolates of the genera *Haloferax* and *Halorubrum* showed the widest inhibitory activity against different archaeal and bacterial strains. Several of the described halocins with broad inhibitory properties are derived from *Haloferax* strains, but halocins of *Halorubrum* producers have not been studied in detail (O'Connor and Shand 2002; Shand and Leyva 2008).

Based on protease sensitivity tests, several of the studied halocins are protein halocins (Table 5) such as halocin H4 (Rodríguez-Valera et al. 1982). The strains with broad inhibitory spectra most probably produce a range of different halocins as demonstrated here for *Haloferax* sp. s5a-1 (strain 12; Table 5). In addition to the protein halocins, some halocins, which were resistant to trypsin and proteinase K, or resistant to trypsin but sensitive to proteinase K, could be microhalocins such as S8 or R1 (O'Connor and Shand 2002). It is considered that halocins are used by microorganisms to compete for nutrients and life space (Torreblanca et al. 1994). However, in a previous study where concentrated hypersaline samples were analyzed for halocin activity, although halocin-producing and sensitive strains were shown to be present, no activity could be detected (Kis-Papo and Oren 2000). This suggests that halocins might not be crucial for interspecies competition of halophiles. The recently introduced rock–paper–scissors model of bacterial antagonism (resistant-sensitive-producer) suggests that the production of antimicrobial substances may promote species diversity in an environment instead of restricting it (Kirkup and Riley 2004). The production of different types of halocins with a wide inhibitory spectrum would be ideal in an extreme environment where the community structure is dominated by prokaryotes. The evidence about the same strain being sensitive to its own halocins (Torreblanca et al. 1994) might suggest that halocins could also be used to control cell density.

The high abundance of halophilic virus–host interactions (Atanasova et al. 2012) as well as the now described halocin production-sensitivity interactions reveal dynamic interplay among these microorganisms which might reflect the worldwide uniformity of hypersaline environments. It is not known whether the viruses infecting the strains and halocins produced by the same strains could influence each other. However, a bacteriocin produced by lactococci is known to induce the lytic cycle of prophages in the lysogenic strains (Madera et al. 2009). In addition, enterococcal bacteriocins have been shown to have antiviral activity against Herpes simplex viruses 1 and 2, affecting the late stages of infection (Wachsman et al. 1999, 2003). Carotovoricins produced by *Erwinia carotovora* are morphologically highly similar to myovirus tails and pyocins of *Pseudomonas aeruginosa* resemble either myovirus or siphovirus tails (Veesler and Cambillau 2011). These bacteriocins also share sequence similarity to the phage tail components and have been suggested to have common ancestry (Yamada et al. 2006; Veesler and Cambillau 2011).

This investigation included close to one hundred archaeal and bacterial isolates from spatially distant sampling places. The high number of production-sensitivity pairs described here suggests that perhaps most of the halophilic organisms carry the potential to produce halocins as we certainly were not able to detect them all. This means, that prokaryotic cells, their viruses, and bacteriocins/archaeosins make up a complex environment where they interact forming an action network in the survival game.
